# Rifaximin ameliorates intestinal inflammation in cirrhotic patients with hepatic encephalopathy

**DOI:** 10.1002/jgh3.12596

**Published:** 2021-06-23

**Authors:** Yasuyuki Tamai, Motoh Iwasa, Akiko Eguchi, Ryuta Shigefuku, Yoshihiro Kamada, Eiji Miyoshi, Yoshiyuki Takei

**Affiliations:** ^1^ Department of Gastroenterology and Hepatology Mie University Graduate School of Medicine Tsu Japan; ^2^ Department of Molecular Biochemistry and Clinical Investigation Osaka University Graduate School of Medicine Osaka Japan

**Keywords:** hepatic encephalopathy, intestinal inflammation, liver cirrhosis, rifaximin

## Abstract

Rifaximin (RFX) treatment can attenuate not only hyperammonemia but also *Enterococcus faecalis* translocation and 10‐7G values, suggesting that RFX treatment may improve intestinal inflammation and result in better overall survival.

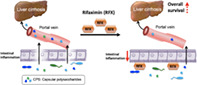

## Introduction

Liver disease affects gut homeostasis, altering intestinal permeability and the gut microbiome proportionally to the degree of liver function impairment as a gut–liver axis.^1^ Dysbiosis, intestinal inflammation, and an impaired intestinal barrier in liver cirrhosis facilitate bacterial product translocation including capsular polysaccharide (CPS) that promotes disease progression *via* immune system activation and subsequent induction of pro‐inflammatory pathways.^1^ CPS is a constituent within the outer layer of many bacterial strains including *Enterococcus* and is the main source of virulence to humans.^2^ Thus, advanced cirrhosis can be seen as the result of an inflammatory syndrome. Recently, we have established novel ELISA systems using glycan antibody 10‐7G mAb, which directly recognizes fucosylated haptoglobin (Fuc‐Hpt) as an index of intestinal inflammation^3^ and using CPS antibody as an index of bacterial product translocation.^4^

Rifaximin (RFX) is an orally administered, non‐absorbed antibiotic that exhibits broad‐spectrum antimicrobial activity within the gastrointestinal tract.^5^ Several studies have reported that RFX reduces plasma ammonia along with the risk of hepatic encephalopathy (HE) and HE‐related hospitalization.^6^ Furthermore, RFX prevents gut inflammation and intestinal barrier impairment in rats.^7^ However, it remains unclear whether RFX has an effect on intestinal inflammation in cirrhotic patients with HE.

The present study aimed to investigate the effect of RFX treatment on intestinal inflammation and reactivity to bacterial product translocation. The changes in 10‐7G as an index of intestinal inflammation, serum CPS to *Enterococcus faecalis* infection as an index of bacterial products, Mac‐2 binding protein (Mac‐2 bp; hepatic fibrosis marker), and routine laboratory data were evaluated.

## Methods

This retrospectively study was approved by the Ethics Committee of Mie and Osaka University. Patients were informed that they could opt out of having their data used. Thirty HE patients were admitted to our institute between February 2017 and October 2018 and were treated with RFX 400 mg three times a day continuously for more than 3 months. All patients had a history of overt HE underlying liver cirrhosis and RFX was administered for the prevention of HE recurrence. Overt HE was diagnosed as per the established West‐Haven criteria. The diagnosis of cirrhosis was based on clinical history, serologic testing, and radiologic imaging. The exclusion criteria were cardiac and/or respiratory failure, renal failure with serum creatinine >2 mg/dL, and clinical or biochemical signs of infection 1 month prior to inclusion. Patients underwent blood tests pretreatment (baseline) and at 3, 6, and 9 months post‐RFX treatment.

Data regarding demographics, clinical characteristics including presence of hepatocellular carcinoma (HCC) and HE, concomitant lactulose and branched‐chain amino acid use, duration, dosage and adverse events of RFX, and number of HE‐related hospital admissions were retrospectively collected from patient hospital records. Blood samples were collected when patients showed up at the hospital, and biochemical examination of blood including albumin and alanine aminotransferase was measured. Albumin–bilirubin (ALBI) score was calculated based on serum albumin and total bilirubin using the following formula: ALBI‐score = (log_10_ bilirubin [μmol/L] × 0.66) + (albumin [g/L] × −0.085). Serum 10‐7G levels (10‐7G values) were determined using a lectin‐antibody ELISA kit.^3^ The presence of infection with *E. faecalis* was evaluated using a CPS‐specific ELISA kit. The serum Mac‐2 bp levels were determined using an ELISA kit (Immuno‐Biological Laboratory, Japan) according to manufacturer's instruction.

Continuous variables are presented as mean ± SD or median (minimum–maximum), and categorical variables are shown as number of patients. A one‐way repeated measures anova was used to compare the measurements among baseline (pretreatment), and at month 3, 6, and 9 of RFX treatment. Relationships between variables were determined using the two‐sided Pearson's correlation coefficient. Receiver operator characteristic (ROC) curves and the corresponding area under the curve (AUC) were used to obtain cutoffs for the outcomes. The Youden index was applied to calculate the optimal cutoff point. Overall survival was measured using the Kaplan–Meier method and compared using the log‐rank test. All statistical analyses were performed using SPSS21.0 software (IBM, Armonk, NY, USA). *P* < 0.05 was considered significant.

## Results

**Figure 1:**
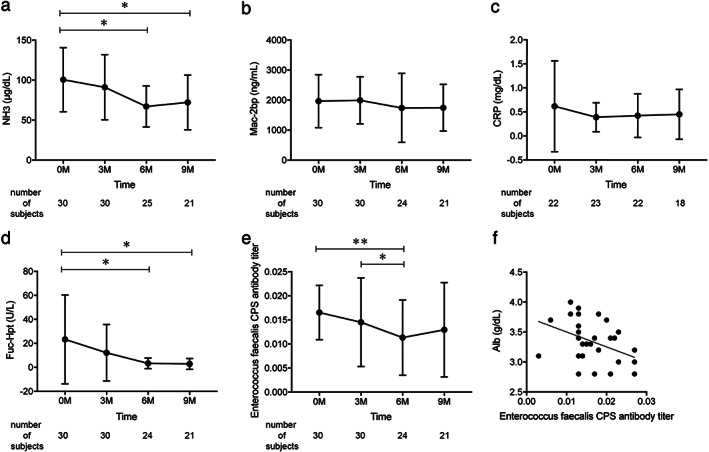
Effect of rifaximin on hepatic function (a–c) and intestinal permeability (d–f). Changes in serum NH_3_ (a), mac‐2 bp (b), CRP (c), 10‐7G antibody titer (d), and *Enterococcus faecalis* CPS antibody titer (e) at baseline and after treatment. (f) Correlation of *E. faecalis* CPS antibody titer with Alb at baseline. **P < 0.01, *P < 0.05. Alb, albumin; CPS, capsular polysaccharide; CRP, C‐reactive protein; NH_3_, ammonia; mac‐2 bp, mac‐2 binding protein.

Patient characteristics are summarized in Table 1. The study cohort consisted of patients with decompensated cirrhosis based on a variety of causative agents including 11 HCV, 3 HBV, 6 NASH, 6 alcohol, and 4 others. The cohort had age 65.5 ± 11.9 (mean ± SD) years, gender 19/11 (male/female), ALBI score −1.92 ± 0.38, and 16 HCC patients. RFX was added to lactulose in 26 cases, and was used in combination with branched‐chain amino acid in 24 cases. RFX treatment significantly reduced venous ammonia levels, especially at 6 and 9 months post‐RFX treatment, when compared with patient baseline measurements (*P* < 0.05; Fig. 1a). We observed no statistically significant differences in the liver fibrosis marker Mac‐2 bp (Fig. 1b) or CRP (Fig. 1c).

**Table 1:** Baseline clinical and biochemical profiles of patients with hepatic encephalopathy

	*n* = 30
Age, years	65.5 ± 11.9
Gender, male/female	19/11
ALBI score	−1.92 ± 0.38
HCC	16
Etiology, HBV/HCV/NASH/alcohol/others	3/11/6/6/4
Alb, g/dL	3.33 ± 0.36
Total bilirubin, mg/dL	1.57 ± 0.83
AST, IU/L	42.0 ± 16.7
ALT, IU/L	26.5 ± 12.7
ALP, U/L	377.6 ± 140.4
NH_3_, μg/dL	100.5 ± 40.1
BUN, mg/dL	19.1 ± 12.0
Creatinine, mg/dL	0.92 ± 0.30
CRP, mg/dL	0.62 ± 0.95
White cell counts, /μL	4482 ± 1948
Hemoglobin, g/dL	11.1 ± 2.0
Platelets, ×10^3^/μL	128.9 ± 74.7
Prothrombin time, %	69.7 ± 17.6
10‐7G, U/L	23.3 ± 37.0
*Enterococcus faecalis* CPS antibody, titer	0.016 ± 0.006
Mac‐2 bp, ng/mL	1984 ± 890

Data are presented as number of patients, mean ± SD, or median (minimum–maximum).

Alb, albumin; ALBI, albumin–bilirubin; ALP, alkaline phosphatase; ALT, alanine aminotransferase; AST, aspartate transaminase; BUN, blood urea nitrogen; CPS, capsular polysaccharide; CRP, C‐reactive protein; HBV, hepatitis B virus; HCC, hepatocellular carcinoma; HCV, hepatitis C virus; Mac‐2 bp, Mac‐2 binding protein; NASH, non‐alcoholic steatohepatitis; NH_3_, ammonia.

The serum values of 10‐7G fell consistently throughout the study period to the point of our observing a statistically significant decrease at 6 and 9 months post‐RFX treatment, when compared with baseline (*P* < 0.05; Fig. 1d). Serum *E. faecalis* CPS antibody titer was also significantly decreased at 6 months post‐RFX treatment (baseline vs. 6 months: *P* < 0.01, 3 *vs* 6 months; Fig. 1e). The baseline CPS levels were negatively correlated with serum albumin levels (*r* = −0.425, *P* < 0.05; Fig. 1f), suggesting that bacterial infection might be associated with hepatic function in cirrhotic patients.

**Figure 2:**
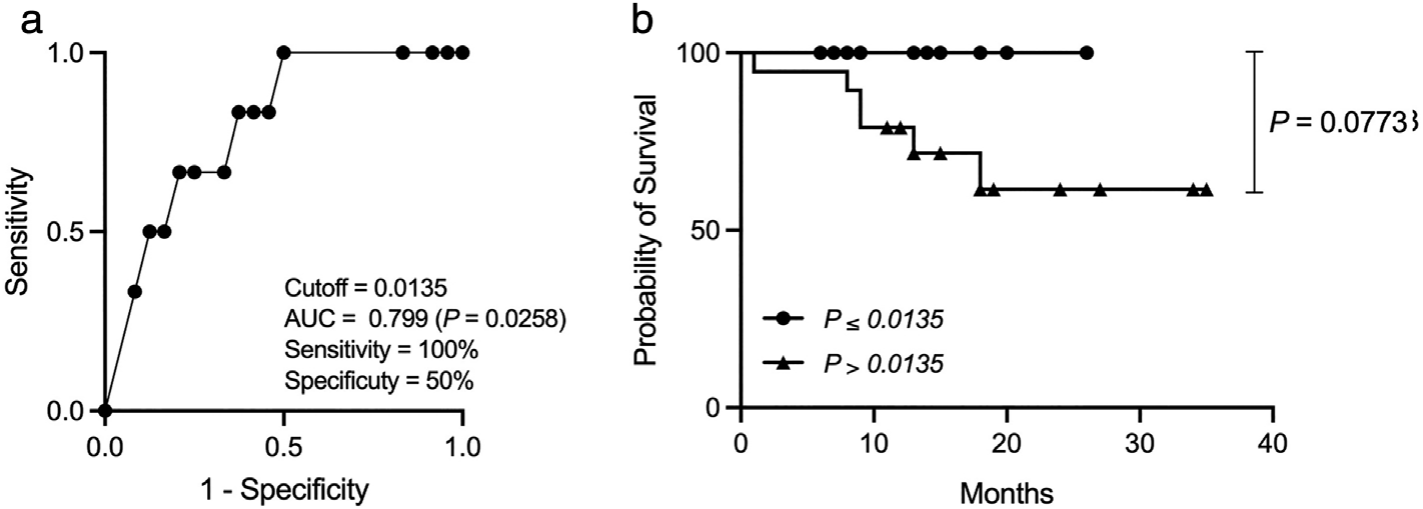
ROC curve for evaluation of overall survival of *Enterococcus faecalis* CPS antibody (a) and survival curve (b). ROC, receiver operator characteristic; CPS, capsular polysaccharide.

There was no discontinuation of RFX treatment owing to intolerable adverse events, and there were six deaths due to a worsening of an underlying disease. ROC analyses concerning predictors of survival yielded AUC values of 0.799 (*P* = 0.0258; Fig. 2a) for *E. faecalis* CPS antibody. We calculated the cutoff value of *E. faecalis* CPS antibody titer at 0.0135 (sensitivity 1.0 and specificity 0.5) from our ROC analysis of survival curves. Patients with low *E. faecalis* CPS antibody titer (<0.0135) showed better overall survival than patients with high *E. faecalis* CPS antibody titer (*P* = 0.0773; Fig.2b).

## Discussion

This study focused on the functional contribution of RFX to the intestinal inflammation in cirrhotic patients with HE. Human zonulin as one of the few known physiological mediators increasing intestinal permeability was identified as prehaptoglobin‐2, which previously had been regarded as the inactive precursor for haptoglobin.^8^ However, widely used commercial zonulin ELISA does not detect precursor of haptoglobin‐2.^9^ In the present study, using our 10‐7G ELISA kit recognizing precursor for haptoglobin, we found that ammonia and 10‐7G values were significantly decreased after RFX treatment in cirrhotic patients with HE. This evidence may suggest an interaction between an improvement in HE and the preservation of intestinal inflammation in RFX‐treated patients.

Liver cirrhosis is associated with profound alterations in gut microbiota and injuries at the defensive mechanisms of intestinal barrier, called liver–gut axis. ^1^, ^10^ Changes in intestinal permeability permit gut‐derived bacterial products including CPS to infiltrate the portal circulation and thus the liver.^11^ In this study, *E. faecalis* CPS antibody titer was significantly decreased by RFX treatment in cirrhotic patients with HE, negatively correlated with serum albumin values at baseline and associated with overall survival. RFX treatment provides potent activity against several species of *Enterococcus* ^6^ and a CPS antibody may be useful in the evaluation and monitoring of reactivity to *Enterococcus* product translocation.

Ammonia hypermetabolism results in astrocyte enlargement and cerebral edema and is thus critically involved in the pathogenesis of HE.^12^ Inflammation appears to be a key driver in the pathogenesis of HE due to the high rate of HE exacerbation in patients who present with concomitant systemic inflammatory response syndrome, and due to the correlation between electroencephalographic findings and blood inflammatory cytokine levels in LC patients.^13^ Our group found no significant association between changes in ammonia level and those in *E. faecalis* CPS antibodies or 10‐7G values. This result would suggest that elevated ammonia, together with inflammation, is intricately and/or independently involved in the onset of HE.

In conclusion, we showed that RFX treatment can attenuate not only hyperammonemia but also *E. faecalis* translocation and 10‐7G values, suggesting that RFX treatment may improve intestinal inflammation.
